# Attempt at Self-Castration

**Published:** 1856-04

**Authors:** J. A. Crounse

**Affiliations:** North Blenheim, N. Y.


### *Attempt at Self-Castration.*

[Communicated for the Boston Medical and Surgical Journal.]

At midnight on the 27th of December last, I received a message to visit, immediately, a man about 30 years of age, married, and having a wife and two children. He is a poor man. When I arrived (within half an hour from the reception of the summons), I found him lying upon the bed, in great pain, and very much distressed. Directing every one to leave the room, and being immediately obeyed, he shocked my ears with the announcement that he had been attempting to castrate himself, but before he succeeded in accomplishing his design, the pain from the rapidly developed inflammation and swelling, caused by his unscientific efforts, compelled him to desist, and to send in great alarm for medical aid, to obviate the mischief he had done. I immediately proceeded to examine the organs, and found the right side of the scrotum laid open throughout its whole length. By exploring with the finger, I found that the testicle was suspended wholly by the spermatic cord, and that it had swollen more rapidly than the integument surrounding it. I immediately determined to make an attempt at saving the testicle, and, with much difficulty, succeeded in bringing the edges of the wound together, which operation required about fifteen sutures, as the testicle, at the time, had increased to more than three times its natural size as compared with its fellow on the opposite side. I employed, immediately after, cold water dressings, and continued these throughout the whole treatment, which occupied about ten days; two or three sutures coming out caused a little gaping, which delayed the cure. On the healing of the wounded scrotum, he was seized with severe pain in his left side, embracing the whole left portion of the chest; there were difficult breathing and cough, amounting to complete pleuritis, which condition lasted three days. The treatment was bleeding, to the extent of relieving the pain, and followed by the usual antiphlogistic means. After a few days' comparative convalescence the pain returned, from cold or some other accidental cause, and extended all the way through, from the left side, over the region of the diaphragm, to the right side. In connection with this pain there were strong evidences of derangement of the liver. Another full bleeding and mercurials, in a few days, brought established convalescence once more. This was again interrupted by a sudden inflammation in the right side, which required another bleeding, and he is at this time, after a period of five weeks, encouragingly convalescent. One thing very remarkable in these shifting inflammatory developments was, that the liver participated very largely in each. The poor victim assigned as his motive for committing this outrage upon himself, that he had heard it was a very simple operation, and would prevent him from having children !

J. A. Crounse.

*North Blenheim, N. Y., Jan.* 21, 1856.

